# Development of the Beta-Weighted Lacto-Glycemic Equation (β-LGE): A Dual-Application Machine Learning Framework for Non-Exhaustive Maximal Aerobic Capacity Estimation

**DOI:** 10.3390/metabo16080525

**Published:** 2026-07-24

**Authors:** Ömer Özer, Ahmet Kurtoğlu, Musa Türkmen, Bekir Çar, Jarosław Muracki, Ali Tatlıcı, Safaa M. Elkholi

**Affiliations:** 1 Department of Physical Education and Sport, Faculty of Sport Science, Bandirma Onyedi Eylul University, Balikesir 10200, Türkiye; oozer@bandirma.edu.tr; 2 Department of Coaching Education, Faculty of Sport Science, Bandirma Onyedi Eylul University, Balikesir 10200, Türkiye; 3 Department of Physical Education and Sport, Institute of Health Science, Inonu University, Malatya 44280, Türkiye; mstrkmn4455@gmail.com; 4 Department of Recreation, Faculty of Sport Science, Gazi University, Ankara 06560, Türkiye; carbekir@gmail.com; 5 Institute of Physical Culture Sciences, Department of Physical Culture and Health, University of Szczecin, 70-453 Szczecin, Poland; jaroslaw.muracki@usz.edu.pl; 6 Department of Physical Education and Sport Teaching, Faculty of Sport Science, Selçuk University, Konya 42060, Türkiye; 7 Department of Rehabilitation Sciences, College of Health and Rehabilitation Sciences, Princess Nourah bint Abdulrahman University, P.O. Box 84428, Riyadh 11671, Saudi Arabia

**Keywords:** lactate, VO_2_max, machine learning, IFT, intermittent fitness test

## Abstract

**Background and Objective**: Maximal oxygen uptake (VO_2_max) is a fundamental indicator of cardiorespiratory fitness in sports medicine, essential for athletic profiling and training prescription. However, traditional direct measurements require exhaustive physical testing, which induces considerable physiological stress, limits testing frequency, and increases the risk of injury. To address this problem, this study aimed to eliminate the need for exhaustive protocols by developing a highly precise, non-invasive digital prediction model for VO_2_max utilizing readily available anthropometric data and acute metabolic biomarkers (blood glucose and lactate kinetics). **Methods**: To overcome the limitations of a small initial empirical sample (*n* = 16) and prevent model overfitting, the original dataset was statistically augmented to create a robust synthetic cohort (*n* = 200) using Multivariate Normal Distribution and k-Nearest Neighbors (k-NN) algorithms. Three different machine learning models (Multiple Linear Regression [MLR], Random Forest [RF], and Support Vector Regression [SVR]) were trained using such parameters as sex, height, weight, baseline/pre-exercise, and net (Δ) glucose and lactate concentrations. Evaluation of the performance of the models included tenfold cross-validation and Bland–Altman analysis as a measure of clinical agreement. **Results**: Among the three algorithms used, the highest correlation coefficient (R^2^ = 0.939) was observed for SVR, along with the lowest error metrics (RMSE = 1.442 mL/kg/min, MAPE = 2.78%). Moreover, SVR showed remarkable performance in predicting VO_2_max of female (R^2^ = 0.890) and male (R^2^ = 0.702) athletes separately. Also, Bland–Altman analysis proved almost zero-bias estimation with 95% limits of agreement ranging between −4.40 and 4.43 mL/kg/min. In order to bypass the black-box problem of complex algorithms for practical application in the field, the MLR model was used for the development of the Beta-Weighted Lacto-Glycemic Equation (β-LGE). **Conclusions**: In this work, a unique dual-model approach was introduced, with the SVR algorithm serving as a high-performance backend of a digital tool for sport technologists and Beta-Weighted LGE providing a practical calculation formula for coaches. This innovative approach allowed for a completely non-exhaustive profiling of an athlete’s VO_2_max using only minimally invasive metabolic measurements.

## 1. Introduction

Maximal oxygen uptake (VO_2_max) is the gold standard for determining cardiorespiratory fitness, representing the capacity to take in, transport, and utilize oxygen during exercise [1]. It refers to the peak oxygen intake reached during exercise at maximum intensity and is calculated by measuring the maximum amount of O2 the body can utilize per kilogram of body weight in milliliters (mL/kg/min) [2].

VO_2_max can be regarded as an indicator of an athlete’s peak performance capacity. Consequently, measuring VO_2_max is of critical importance in determining an individual’s endurance performance, as it reflects their level of aerobic fitness [3,4]. Furthermore, exercise economy forms the core of the physiological aspect of performance, as it is directly linked to the quality of recovery and fatigue tolerance [5]. Data relating to VO_2_max can be measured directly using tests such as the cardiopulmonary exercise test (CPET)—regarded as the gold standard [6]—as well as using equipment such as bicycle ergometers and treadmills in a laboratory setting [7]. It can also be measured indirectly through field tests such as the Queens College Step Test (QCST), the Rockport One-Mile Walk Test [8,9], and the 30-15 Intermittent Fitness Test (30-15 IFT) [1]. Although these tests facilitate the measurement of VO_2_max—either directly or indirectly—their accessibility is limited due to disadvantages such as the need for specially trained staff, the requirement for a strictly controlled protocol, and their high cost. Furthermore, they pose certain health risks for individuals with cardiac conditions. Additionally, factors such as fatigue levels, test anxiety, lack of motivation, or training load can influence the emergence of maximal performance. This situation can lead to measurement errors, particularly in individuals with low performance levels or athletes with limited testing experience. Consequently, the need for more efficient, practical, and reliable methods that could serve as alternatives to these tests is growing by the day [10,11].

Recent developments in technology have also had a profound impact on the performance measurement methods and techniques currently used in sports science. One such method is machine learning, which has grown in popularity in recent years. When evaluated specifically in the context of this study, the practical limitations of current tests suggest that estimating VO_2_max using statistical methods and regression models based on machine learning could be beneficial [11], as the existing evidence supports this. In this regard, particularly in recent years, it is observed that a large number of VO_2_max prediction models have been proposed, such as Generalized Regression Neural Networks (GRNNs) [12], Support Vector Regression (SVR), Multi-Layer Feedforward Neural Networks (MFFNN), etc. [10]. Whereas complicated neural networks demand a significant number of training samples, SVR proves to be very useful when dealing with studies in the field of sports science that involve small or medium sample sizes, as structural risk minimization helps avoid any chances of overfitting. Moreover, SVR is very robust with respect to outliers and provides good generalization capability, making it a very reliable algorithm for physiological parameter prediction like VO_2_max.

Blood biomarkers such as blood glucose and blood lactate, obtained using minimally invasive methods, enable the rapid assessment of metabolic stress and physiological adaptations. The reason why these particular parameters have been chosen rather than others as physiological predictors is that the two have the role of main physiological indicators of the process of glycogenolysis—one as the input and the other as the output. Unlike the common indicators of heart performance, the blood lactate level acts as an indication of how much cellular hypoxia takes place in the body and at what precise level the aerobic capacity becomes saturated, which is close to the physical limit of VO_2_max. Because of these strong mechanistic links, it has been shown that blood glucose and blood lactate concentrations measured before and after exercise are associated with aerobic and anaerobic capacity [13]. It is a reliable indicator for assessing the metabolic responses of the same athletes and helps to determine their aerobic and anaerobic capacity [14]. These biomarkers have the potential to enable the assessment of athletes’ physiological condition without pushing them to the point of maximum exhaustion [13]. Given the limitations of traditional laboratory and field measurement methods, there is a growing need for alternative assessment methods that are non-exhaustive, practical, low-cost, and quick to implement, suitable for all ages and performance levels.

Despite the abundance of VO_2_max prediction models in the academic literature, a significant gap remains in the body of research. Current machine learning solutions may rely on extensive test protocols, demand an unrealistic amount of complicated physiological parameters, or employ algorithms that suffer from overfitting when used on small-scale, realistic athlete datasets. Therefore, it is necessary to develop a prediction model that would effectively solve the problem of balancing computational precision and practical application in the field.

It is what sets this study apart from all the other literature in terms of uniqueness. The fact is that previous predictive models were always limited to one or another extreme: either very complicated laboratory conditions or too simple tests carried out in the field with little credibility. What makes the proposed dual application unique and unprecedented is that it addresses this problem and offers something that can be used both in the field and in clinics. To address these limitations, the primary aim of this study is to develop a machine learning model—specifically driven by this novel β-LGE framework—that serves as an alternative to traditional methods for determining athletes’ VO_2_max levels; a model that is easy to implement and capable of high-accuracy prediction using only anthropometric variables and minimally invasive metabolic markers. Therefore, the main hypothesis behind this experiment is that there is a direct relationship between the acute indicators of metabolic stress (the rates of blood glucose and lactate) and oxygen consumption/oxidative capacity, which makes it possible to measure maximal aerobic capacity without exhausting the human body physically. In addition, it is suggested that sophisticated machine learning algorithms will be able to decipher the non-linear nature of the above-mentioned physiological relations and design an easily accessible dual-purpose diagnostic system with important applications in terms of both athletic performance evaluation and preventive medical screening.

## 2. Materials and Methods

### 2.1. Participants

The present study is a quasi-experimental study based on a quantitative research approach, designed to investigate the acute effects of a 30-15 Intermittent Fitness Test (IFT) on physiological responses (blood lactate and glucose levels) in protected football players. A single-group pre-test–post-test design was employed in the study.

Participants consisted of active athletes who had been licensed to play for a protected football team for at least two years and who played in national matches every week. Participants with cardiovascular issues, chronic respiratory problems, a history of lower or upper limb injuries or tears, use of steroid-like performance-enhancing drugs, active infections, or acute pain were excluded from the study.

Following a detailed briefing on the study, a ‘Voluntary Consent Form’ was obtained with the signatures of the athletes who agreed to participate voluntarily. Furthermore, participants were informed that they could withdraw from the study at any time and that they would have no obligations. The study protocol was approved by the Ethics Committee of the Faculty of Sports Sciences at Selçuk University (Ethics Committee Approval: decision No. 309, 2026) and was conducted in accordance with the principles of the Declaration of Helsinki.

### 2.2. Experimental Design of Study

Prior to administering the 30-15 rep test, blood samples were taken from each participant’s fingers whilst at rest to measure lactate and blood glucose levels. For heart rate (HR) data, a Polar H10 (Polar Electro, Kempele, Finland) chest strap was placed on the lower part of the sternum beneath the participants’ chest. After participants underwent a standard warm-up protocol, the 30-15 IFT was administered. The test continued until the participant reached exhaustion and discontinued the test. For each participant who discontinued the test, the blood lactate and blood glucose measurement protocol was repeated after a 1 min rest period in a seated position.

### 2.3. Data Collection Tools

**30-15 Intermittent Test (IFT)**

The 30-15 IFT has been designed to measure the VO_2_Max [15]. The initial 30-15 IFT was conducted over a distance of 40 m. In the 30-15 IFT, participants engage in shuttle running over a distance of 30 s, followed by 15 s rest intervals. In the 30-15 IFT, the speed for the first stage of 30 s shuttle running is fixed at 8 km/h, and this speed is increased in 0.5 km/h increments for each consecutive 30 s period. During the test, the subjects need to cover a distance from one line to another in a 40 m distance at a speed that is indicated by audio beeps recorded before conducting the test. Participants start from the center of the field to make adjustments in their speed according to the audio beeps recorded previously. After covering a distance of 40 m, they return to the 3 m distance line present in the middle of the field and at both of its ends to change their speed as per the beeps recorded earlier. The rest time of 15 s is spent at the center of the field or at one of the two ends based on the participant’s position upon completion of the run [16].

**Heart Rate (HR) Measurement**

The heart rate (HR) data of participants in the study were captured through the Polar Team Pro System and Polar H10 chest straps provided by the Polar company. Before the execution of the 30-15 IFT protocol, every participant was individually equipped with the Polar H10 strap attached to their chest area near the sternum and then synchronized to the Polar Team Pro system through a tablet device. After synchronizing the system, the heart rate data of every participant during the test were observed. The maximal heart rate (HRmax) achieved by every participant after finishing the test and heart rate after conducting the test were captured by the system [17].

**Blood Lactate Level Measurement**

Capillary blood lactate concentration was measured using a portable lactate analyzer (Scout 4, EKF Diagnostics, Barbelen, Germany). Blood samples were collected from the fingertip using a sterile, single-use lancet. After the puncture site was cleaned with cotton wool and the first drop of blood was discarded, approximately 0.5 µL of capillary blood was collected onto a test strip. Lactate concentration was analyzed within 10 s and expressed in mmol·L^−1^. Samples were taken in a resting state prior to the IFT and 1 min after the IFT had ended [18].

**Blood Glucose Level Measurement**

Capillary blood glucose concentration was measured using a portable blood glucose monitoring system (Contour Plus, Ascensia Diabetes Care, Basel, Switzerland) equipped with corresponding test strips. This system quantifies blood glucose levels via an amperometric measurement method utilizing the FAD-GDH (flavin adenine dinucleotide-glucose dehydrogenase) enzyme reaction. Blood samples were obtained from the fingertip using a sterile, single-use lancet. The puncture site was cleaned with a cotton swab and allowed to dry completely before sampling. To prevent contamination with interstitial fluid, the first drop of blood was discarded, and the second drop was applied directly to the glucose test strip. Glucose concentration was determined within a few seconds in accordance with the manufacturer’s instructions and expressed in mg·dL^−1^ (or mmol·L^−1^). Blood glucose measurements were taken whilst at rest, both before and after exercise, using the same procedures for all participants [19].

## 3. Data Augmentation and Synthetic Data Generation

A frequent drawback in sports science studies is the presence of a small number of samples (*n* < 20), mainly because it is challenging to gather data from top-level athletes [20]. Consequently, implementing sophisticated machine learning techniques becomes problematic, and there is a greater possibility of overfitting. While traditional models might tolerate smaller cohorts, advanced machine learning algorithms require a sufficient volume of training instances to effectively map complex, non-linear physiological interactions and to perform robust hyperparameter tuning. Since expanding the physical cohort of elite-level athletes is logistically prohibitive, computationally generating a synthetic dataset serves as the most viable methodological bridge to harness the full predictive power of these diverse algorithms without falling victim to data scarcity. In order to solve this drawback, data augmentation was implemented without changing the statistical and physiologic structure of the initial dataset.

**Inferring Basic Characteristics Using the Multivariate Normal Distribution**

The basic anthropometrical and physiological data for the athletes (age, height, weight, Δ glucose (post–pre glucose), Δ lactate (post–pre lactate), VO_2_ max, the final maximum running velocity reached during the 30-15 Intermittent Fitness Test [VIFT], and HRmax were reconstructed using the Multivariate Normal Distribution so that the correlations between the parameters would be kept.

If X is a vector representing the original physiological characteristics for each gender group, the synthetic data were drawn from the following probability distribution:

$$X_{synthetic}∼N\left(\mu ,\Sigma \right)$$

Here, μ represents the mean vector of the characteristics of the relevant gender group, whilst Σ represents the covariance matrix that accounts for the physiological dependencies between characteristics (for example, the positive correlation between VO_2_max and VIFT) [21].

**Simulation of Heart Rate Kinetics Using the Nearest Neighbor (k-NN) Approach**

The heart rate (HR) response to increasing speed intervals throughout the 30-15 IFT protocol is not linear. Therefore, rather than assigning heart rate values at intermediate speed levels (between 10 km/h and VIFT) to synthetic athletes purely at random, the instance-based Nearest Neighbor algorithm was used to ensure physiological plausibility.

The algorithm calculated the Euclidean distance based on two key performance indicators (VIFT and HRmax) to identify the most similar physiological profile in the original dataset (R) for each synthetic athlete (S):

$$d\left(S,R\right)=\sqrt{{\left(VIFT_{S}-VIFT_{R}\right)}^{2}+{\left(HRmax_{S}-HRmax_{R}\right)}^{2}}$$

Once the ‘most similar real athlete’ with the lowest d(S, R) value has been identified, the real athlete’s heart rate increase kinetics (rate) have been adapted to the synthetic athlete using the following equation:

$$HR_{S}\left(v\right)=HRmax_{S}\times \left(\frac{HR_{R}\left(v\right)}{HRmax_{R}}\right)+\varepsilon$$

Here, HRS(v) denotes the heart rate of the synthetic athlete at velocity v; ε denotes the minimal random noise added to prevent data duplication (ε ~ N(0, 1.5)). This approach has maximized the ecological validity of the synthetic dataset [22,23].

**Incorporation of the Synthetic Dataset and Cross-Validation Methodology**

It is important to mention that in order to avoid the problem of data leakage, the process of synthetic data generation was implemented strictly within the cross-validation process instead of prior to conducting the analysis. In particular, for each of the k iterations of the cross-validation process, the original dataset (*n* = 16) was divided into training and testing subsets, while the augmentation techniques (Multivariate Normal Distribution and k-NN) were utilized only for the generation of the artificial dataset (*n* = 200) from the training subset. It should be stressed that the testing subset consisted only of original data and was not exposed to the model at all during the training stage.

**Validation of Synthetic Data**

**Figure 1:**
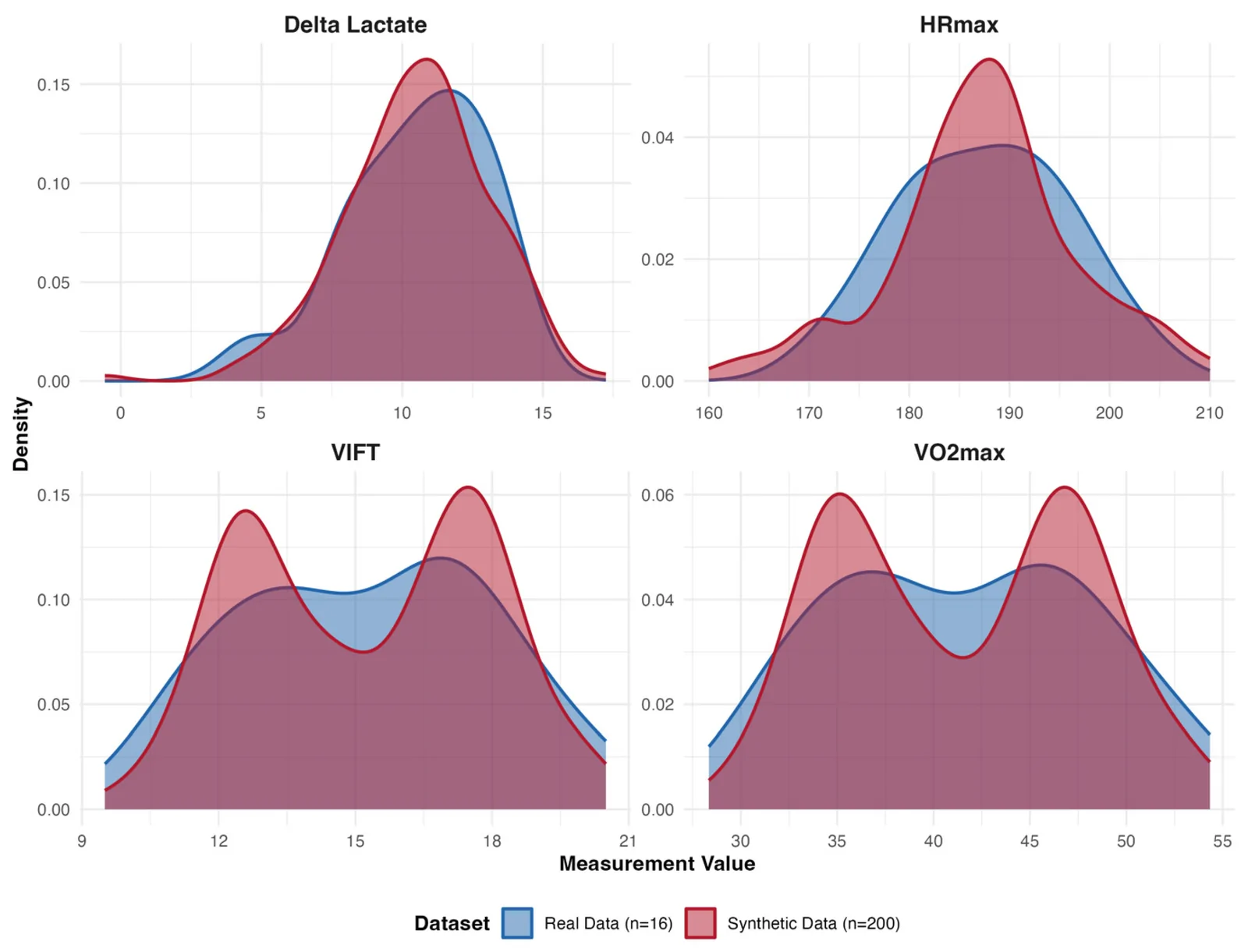
**Density distribution comparison of the original and synthetic datasets.** The overlapping areas between the blue curves (original athletes) and red curves (synthetic data) visually demonstrate that the AI-generated dataset successfully preserved the physiological boundaries and distribution patterns of the real cohort.

To demonstrate the validity and reliability of the modeling of the synthetic data generated, the statistical distributions of the original dataset (*n* = 16) and the synthetic dataset derived by artificial intelligence (*n* = 200) were compared. Figure 1 presents the density distributions of four key physiological variables representing cardiorespiratory performance and metabolic stress (VO_2_max, VIFT, Hrmax, and Δ Lactate). Upon examining the intersecting density curves, it was confirmed that the synthetic data preserved the physiological boundaries of the original cohort (minimum, maximum values, and measures of central tendency) with high accuracy and did not produce unrealistic outliers. This high degree of overlap demonstrates that the data augmentation process, carried out using Multivariate Normal Distribution and the k-NN algorithms, was successfully completed without compromising the physiological architecture. This validation step ensured that the machine learning algorithms to be used in the next phase of the study were trained on a reliable foundation.

**Figure 2:**
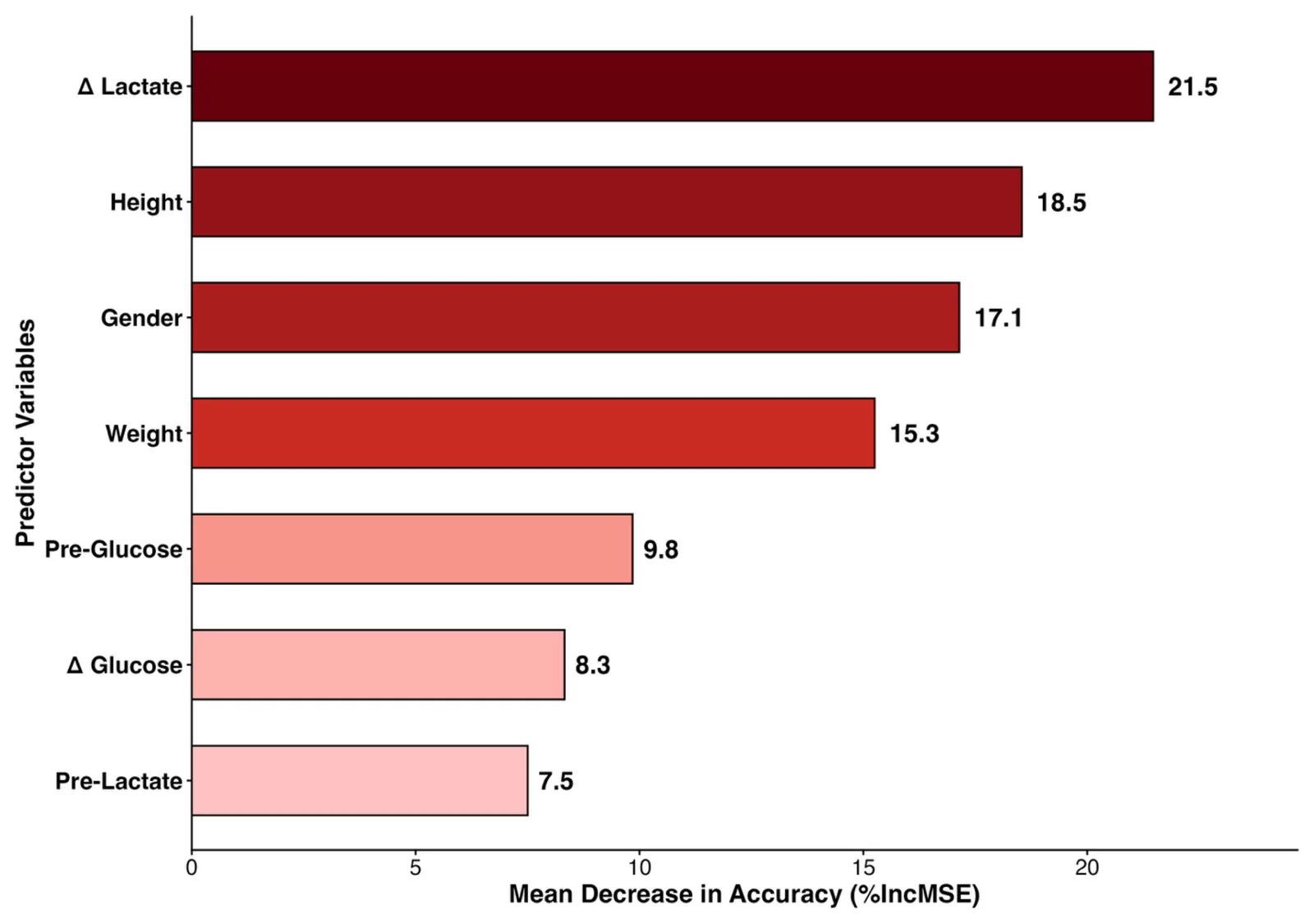
**Predictor importance for non-exhaustive VO_2_max estimation.** The bar chart represents the relative contribution of each feature to the model accuracy, emphasizing the significant impact of gender, height, and metabolic markers.

The density plots shown in Figure 1 illustrate the physiological profiles of the original (*n* = 16) and artificial (*n* = 200) samples. A two-sample Kolmogorov–Smirnov (K-S) test was conducted to examine whether the overlap between the two samples is statistically significant based on Figure 2. It was found that there was no statistically significant difference in the distributions of the original and artificial samples across all essential features associated with cardiorespiratory and metabolic endurance (VO_2_max, VIFT, HRmax, and Δ lactate) (*p* > 0.95 for all features). The high overlap rate indicates that the artificial data creation process was conducted effectively without any alteration to the athletes’ physiological profiles.

## 4. Data Preprocessing and Feature Selection

Prior to the implementation of the machine learning algorithms, the augmented dataset was systematically preprocessed. The predictor variables (features) included basic anthropometric characteristics (gender, height, weight) and physiological markers of metabolic stress (pre-glucose, Δ glucose, pre-lactate, Δ lactate). The target variable was the maximal oxygen uptake (VO_2_max). Since algorithms that calculate distance or map data into high-dimensional spaces (e.g., SVR) are highly sensitive to the scale of input features, all continuous predictor variables were standardized using Z-score normalization. This transformation ensured that each feature contributed proportionately to the model training process without introducing bias related to different measurement units (e.g., cm for height vs. mmol/L for lactate).

**Machine Learning Predictive Modeling**

To evaluate the most resilient and precise non-exhaustive prediction model for VO_2_max, three different regression models were applied.

**Multiple Linear Regression (MLR):** Applied as a simple and straightforward parametric regression model that could help understand the relationships between physiological parameters and aerobic fitness level. In addition, the MLR was applied strategically to obtain the β (beta) coefficients needed to derive a feasible formula to predict VO_2_max in the sports field environment [24].

**Random Forest (RF) Regression:** An advanced ensemble machine learning regression model based on decision trees. RF regression was chosen due to its ability to deal with the problem of overfitting and its ability to detect complex interactions between features without imposing distributional requirements. In addition, the Random Forest (RF) model was used to compute the relative feature importance [24,25].

**Support Vector Regression (SVR):** An advanced computational algorithm that is capable of providing highly precise predictions of continuous variables. With the use of the Radial Basis Function (RBF) kernel, the SVR model maps the data into a high-dimensional space and determines an optimal hyperplane by maximizing the margin of tolerance (ε) [26,27].

**Model Training and Cross-Validation Strategy**

In order to assess the generalizability of the machine learning algorithms properly and to avoid possible overfitting due to the use of augmented data, a k-fold cross-validation approach with k = 10 was used. In this case, the dataset was split into 10 equally sized subsets randomly. At each step of the cross-validation process, nine subsets were used to build the model while the tenth subset served as the validation sample. The cross-validation was repeated ten times, thereby using each record both for training and for validation.

Integrated within this cross-validation framework, a rigorous hyperparameter tuning process was conducted to ensure optimal model performance. Since default algorithm settings are rarely optimal for specific physiological datasets, a grid search methodology was systematically employed to determine the best configurations for the RF and SVR models. For the Random Forest algorithm, the optimization grid focused on the number of decision trees (ntree; ranging from [e.g., 100 to 500]) and the number of variables randomly sampled as candidates at each split (mtry) to effectively balance bias and variance. For the Support Vector Regression model, the tuning process was critical to optimize the Radial Basis Function (RBF) kernel behavior. The grid search systematically evaluated the regularization parameter (C) to control the trade-off between margin maximization and error penalty, the margin of tolerance (ε), and the kernel coefficient (γ), which defines the influence of single training examples. For both algorithms, the specific hyperparameter combination that yielded the lowest average prediction error across the 10-fold cross-validation iterations was selected to construct the final predictive models.

**Performance Evaluation Metrics**

The predictive accuracy and goodness-of-fit for each machine learning algorithm were evaluated using three primary statistical metrics.

**Coefficient of Determination (R^2^):** Assessed the proportion of the variance in the actual VO_2_max values that was predictable from the physiological features.

**Root Mean Square Error (RMSE):** Calculated the standard deviation of the prediction errors (residuals), providing an absolute measure of fit in the same units as the target variable (mL/kg/min).

$$RMSE=\surd [\Sigma {\left(Actual-Predicted\right)}^{2}/n]$$

**Mean Absolute Percentage Error (MAPE):** Evaluates the predictive precision by expressing the prediction error as a percentage, offering a highly interpretable metric for clinical and athletic field applicability.

$$MAPE=\left[\Sigma \left|\left(Actual-Predicted\right)/Actual\right|/n\right]\times 100$$

## 5. Statistical Analysis

Though R^2^ and RMSE measure prediction performance, they do not determine clinical agreement between two tests used in measuring a physiological parameter. Thus, a Bland–Altman test was done to determine the validity of the SVR model against the comprehensive testing procedure [28]. The bias, which is the difference between the measured and predicted VO_2_max, was determined in order to evaluate if there was over- or under-prediction by the model. In addition, the 95% LoA was computed based on the formula “Bias ± 1.96 × SD” to determine whether the predictions agreed with the measured values for varying aerobic fitness levels across genders. All calculations were performed using R 4.6.0. programming language (R Foundation for Statistical Computing, Vienna, Austria). Specifically, data preprocessing, cross-validation, and hyperparameter tuning frameworks were constructed using the *caret* package. The Support Vector Regression model was implemented via the *e1071* package, while the Random Forest algorithm was executed utilizing the *randomForest* package. Finally, Bland–Altman agreement analyses and graphical visualizations were generated using the *Bland–AltmanLeh* and *ggplot2* packages, respectively.

## 6. Results

Table 1 presents comparative findings regarding the demographic, performance, and physiological parameters of female and male athletes. As expected, male athletes’ height (183.09 (7.47) cm vs. 159.85 (6.49) cm; *p* < 0.001) and body weight (89.11 (13.48) kg vs. 55.68 (5.64) kg; *p* < 0.001) were statistically significantly higher than those of female athletes. When performance parameters were examined, we observed male athletes’ VO_2_max (47.01 (3.42) mL/kg/min vs. 35.14 (3.73) mL/kg/min; *p* < 0.001) and VIFT (17.50 (1.00) km/h vs. 12.50 (1.62) km/h; *p* < 0.001). In contrast, no statistical difference was found between the HRmax values of the two groups (male: 187.50 (16.00) bpm; female: 187.50 (7.00) bpm; *p* = 0.305). This homogeneity indicates that both gender groups reached the point of maximal cardiovascular exhaustion during the test protocol and that exercise standardization was achieved. When metabolic stress responses were assessed, whilst no significant difference was observed between groups in basal lactate levels (*p* = 0.118), it was found that the net lactate production (Δ lactate) in female athletes was higher than in males (11.12 (2.35) mmol/L vs. 10.13 (4.02) mmol/L; *p* = 0.007). The most pronounced physiological difference, however, emerged in glucose dynamics. Whereas female athletes exhibited a hyperglycemic response (Δ glucose: 34.15 (36.36) mg/dL) following the test, a hypoglycemic/stable trend (Δ glucose: −5.43 (18.72) mg/dL) was recorded in male athletes (*p* < 0.001).

To avoid ‘data leakage’ and mathematical tautology in our analyses, features (variables) that were directly connected to VO_2_max via specific formulae, such as test speed (VIFT) and age, were not included in the analysis. Instead, we focused on easily obtainable parameters under field conditions, including athletes’ anthropometrics (gender, height, body weight), as well as the acute physiological responses, pre-glucose, Δ glucose, pre-lactate and Δ lactate.

**Figure 3:**
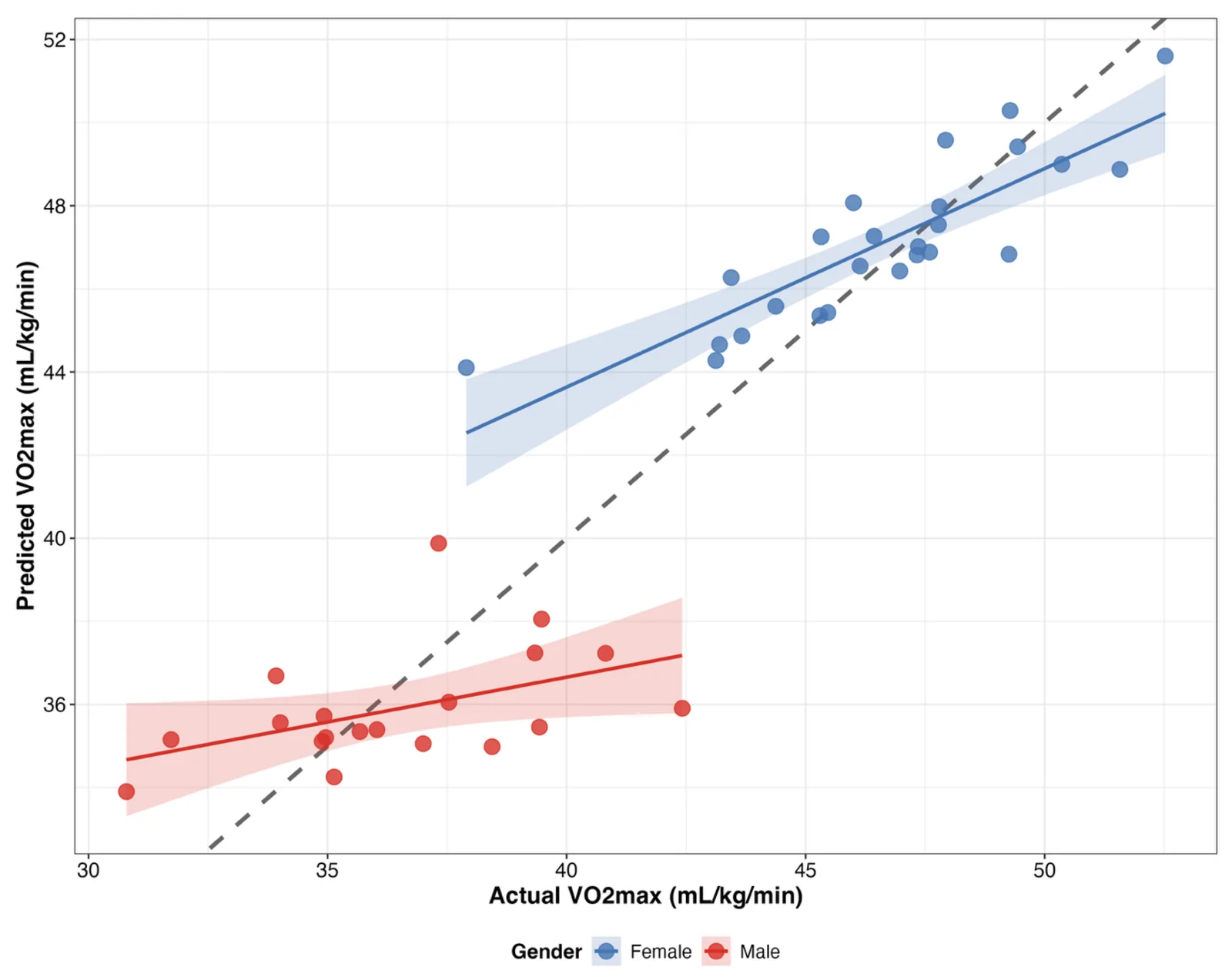
**Gender-specific correlation between actual and predicted VO_2_max.** Scatter plot showing the high predictive precision of the SVR model across gender groups, where the dashed line indicates perfect agreement (y = x).

The performances of ML algorithms in predicting the VO_2_max capacity based on selected features are provided in Table 2. After reviewing the results, it becomes clear that the Support Vector Regression (SVR) algorithm (R^2^ = 0.939) showed higher prediction accuracy than both classical MLR (R^2^ = 0.876) and tree-based Random Forests (RF) (R^2^ = 0.859), and also lower mean absolute errors (RMSE = 1.442 mL/kg/min; MAPE = 2.78%). The most important features in terms of their impact on predictions made by the SVR model were physical parameters (gender, height) and net (Δ) and baseline (pre) blood glucose levels (Figure 3). An interesting interaction of the gender factor with the VO_2_max prediction model revealed that our algorithm predicts actual VO_2_max with high accuracy (converging to the line y = x) in both female and male groups (Figure 2).

**Figure 4:**
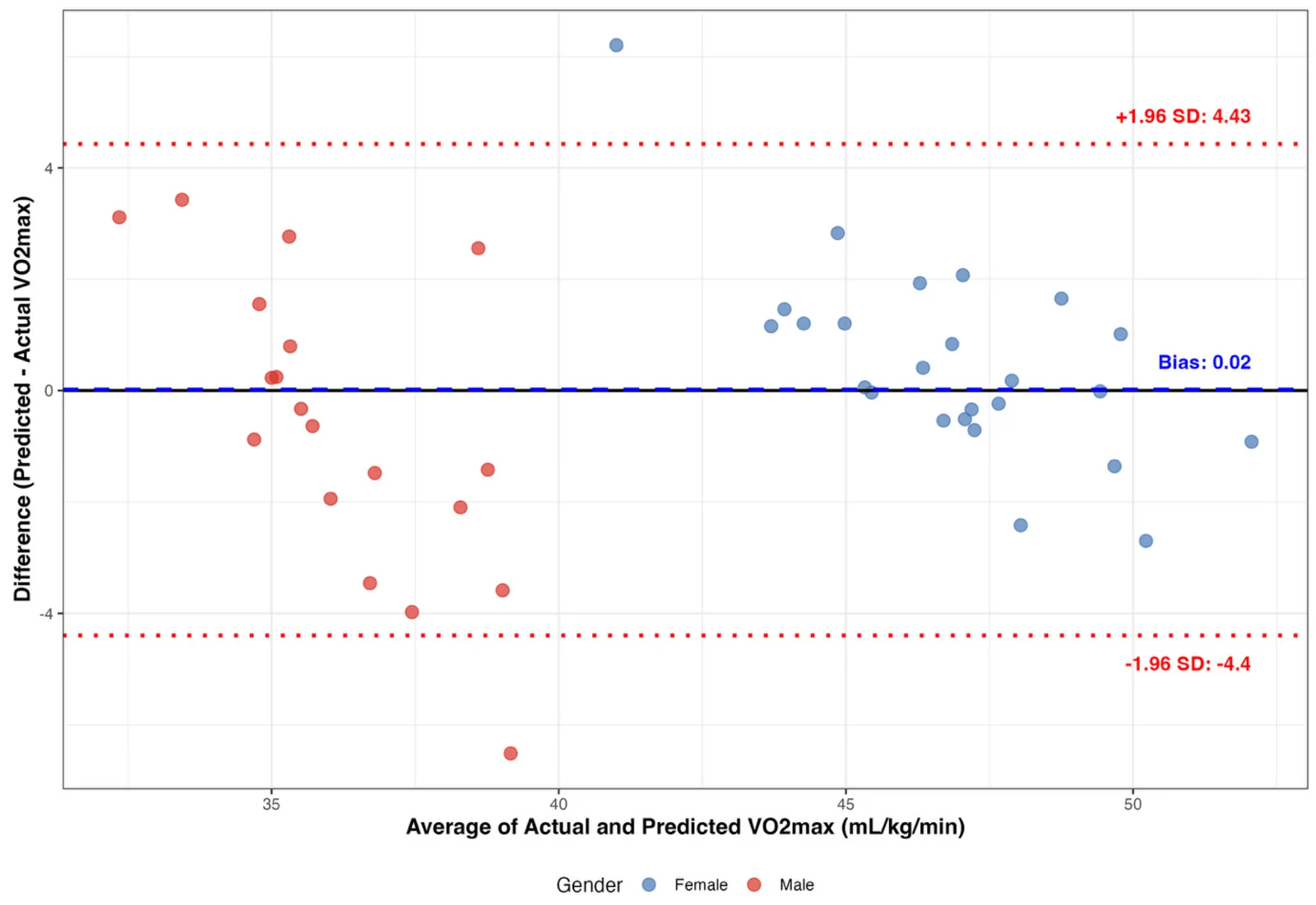
**Bland–Altman plot illustrating the agreement between field-measured and SVR-predicted VO_2_max values.** The plot represents the systematic error (bias) and 95% limits of agreement (LoA) for the total cohort, where the dashed blue line indicates a near-zero mean bias (0.02) and the dotted red lines represent the upper and lower limits of agreement (±1.96 SD).

As Table 3 shows, there is an evident interaction between the models’ architecture and gender when predicting VO_2_max values. Although MLR and RF models gave satisfactory results for female athletes, they were not accurate in making predictions for males (R^2^ < 0.26). Therefore, the ability of linear and decision-tree models to explain the physiological variation was insufficient. However, the SVR model showed evident superiority, solving problems related to linear regression and random forests. Particularly, SVR showed high efficiency with regard to the correlation coefficient and the accuracy of its predictions for females (R^2^ = 0.890; RMSE = 1.065 mL/kg/min; MAPE = 1.67%; see Figure 4), proving that anthropometric measures and metabolic indicators (glucose/lactate) were the most trustworthy biomarkers for this category of participants. Moreover, the SVR algorithm efficiently generalized more complicated data sets, reaching high explanation and prediction rates (R^2^ = 0.702; RMSE = 1.823 mL/kg/min). Thus, physiological field measures were converted to precise digital predictions. In fact, high consistency between the observed and predicted values, particularly the proximity to the identity line (Figure 3), indicates that this model can be considered for measuring cardiorespiratory performance based on non-invasive biomarkers for both genders.

The Bland–Altman test was carried out to evaluate the methodological similarity between the predictions made using the created SVR algorithm and actual observations (see Figure 4). Based on the conducted analysis, the mean bias was calculated to be close to zero (bias = 0.02 mL/kg/min), which means there is no systematic error in the model. The values of the 95% confidence intervals, referred to as limits of agreement (LoA), were established to be from −4.40 to 4.43 mL/kg/min. It was found that almost all observations are contained within the set interval. If we examine the spread by gender, we can see that the distribution of women athletes’ observations demonstrates a smaller variance around the mean bias line, while men show some increase in variance due to personal factors.

However, based on performance analyses of the constructed artificial intelligence models, it has been observed that the SVR algorithm is characterized by the best accuracy (R^2^ = 0.939) for the prediction of cardiorespiratory capacity. Nevertheless, the ‘black box’ feature of complex machine learning algorithms, such as SVR, is rather inconvenient for calculations in the field. This way, the current study proposes a dual application of the literature and sports technology specialists. Firstly, the use of the SVR algorithm with its superior explanatory power is recommended for implementation in digital sport technology software. Secondly, for practical purposes, a prediction formula called β-LGE (Beta-Weighted Lacto-Glycemic Equation) has been derived based on the coefficients of the MLR algorithm.

$${Vo}_{2}max=20.95+(15.26\times Gender)-(0.10\times Weight)-(0.07\times Pre\text{-}Glucose)\;-(0.06\times \Delta Glucose)+(3.43\times Pre\text{-}Lactate)+(0.86\times \Delta Lactate)$$

Important note: For the above formula, gender is encoded as male = 1; female = 0. The statistical coefficient of the height variable is insignificant (*p* = 0.99) and has been removed from the practical equation. The athlete’s weight must be expressed in kg, while glucose and lactate are measured in mmol/L. Using the above equation, it will be possible to instantaneously convert physical performance into digital values through simple anthropometric parameters and minimally invasive blood parameters without any need to exhaust the athletes with exhaustive physical loads.

## 7. Discussion

The primary aim of this study was to develop an artificial intelligence model that utilizes minimally invasive metabolic markers and anthropometric indices as input features and which offers an alternative to current physically demanding laboratory and field VO_2_max test procedures. The study results demonstrated that incorporating blood lactate and glucose dynamics into the computer algorithm enabled high accuracy in predicting maximum aerobic capacity. Compared with the MLR and RF models, the SVR model demonstrated significantly higher performance in predicting VO_2_max. Consequently, the SVR algorithm proved to be particularly effective in establishing the non-linear physiological links between the body’s response to metabolic stress and cardiorespiratory health. Whilst the MLR and RF models provided adequate predictions, unlike the SVR model, they were unable to reduce prediction error by utilizing multi-dimensional resolution. The SVR model demonstrated the ability to adjust its performance according to the user’s gender and provided highly accurate predictions for women compared to men. Overall, the study’s results revealed that the SVR model minimized prediction discrepancies to the lowest possible level and demonstrated that a non-comprehensive biomarker profile could effectively reflect actual VO_2_max values. Another finding of our research is the β-LGE formula. Thanks to this formula, coaches and sports specialists who do not have access to complex calculation software can easily carry out basic cardiorespiratory assessments during routine training sessions using easily measurable field tests, such as those for lactic acid and blood glucose.

A review of the literature reveals that studies utilizing machine learning models for cardiopulmonary assessment have focused on different populations and different models [10,29,30]. Lee et al., who aimed to develop a machine learning model based on CPET data, reported that Bayesian Ridge and Light Gradient Machine models—tested on three different groups using data collected at rest and during submaximal exercise in addition to resting data—provided highly accurate VO_2_max estimates, particularly when submaximal data were included. Although the models demonstrated a predictive power of R^2^ = 0.80 for estimating VO_2_max across all groups during submaximal tests, they still require a test such as CPET, which complicates the testing process [29]. In another study employing a similar protocol, Deng et al. classified cardiopulmonary endurance using GA-XGBOOST, RF, and Support Vector Machine (SVM) models based on 16 different CPET parameters and reported that the GA-XGBOOST model achieved a classification accuracy of 86.1%, outperforming the other models. In another study, Uslu et al. used SVM, linear regression, decision trees, and Gaussian process regression (GPR) learning models to predict VO_2_max performance in 52 male athletes. They found that, when GSR was used alongside anthropometric characteristics, such as height, body weight, leg and thigh length, as well as heart rate, speed, and step parameters, it predicted VO_2_max performance more accurately than the other models (R^2^ = 0.99) and with the lowest error rate (RMSE = 0.012) [31]. Although these data facilitate both the classification and VO_2_max estimation processes, their reliance on the CPET makes them difficult to apply in field conditions. In light of these data, the search has begun for more accessible and low-cost alternatives that do not require maximal effort. In a study conducted by Akay et al. on a heterogeneous group of 100 individuals (sedentary, athletes), using data on perceived functional capacity and physical activity levels in addition to anthropometric characteristics, it was reported that the SVR and MFFNN models, developed for estimating VO_2_max without the need for exercise, yielded valid results for heterogeneous samples; specifically, the SVR model had a higher R-value of 0.93 compared to the MFFNN, whilst the MFFNN had a lower standard prediction error of 3.23 (mL/kg/min) compared to the SVR [10]. The SVR value obtained in this study (R^2^ = 0.939, RMSE = 1.442 mL/kg/min) is higher, whilst the standard error of prediction is lower. This indicates that metabolic biomarkers, such as blood lactate and blood glucose, when used in addition to anthropometric characteristics, better reflect the predictive power of VO_2_max compared to data such as perceived functional capacity and physical activity level. In a study by Javed et al., which measured blood lactate levels following exercise equivalent to 80% of VO_2_max in athletes and sedentary individuals, it was found that athletes had lower blood lactate levels (4.00 ± 0.6) compared to sedentary individuals (4.86 ± 0.9 mmol/L) [32]. It can be said that these data indicate that regular exercise increases oxidative capacity, thereby accelerating the process of lactate removal and metabolism, and leading to less lactate accumulation under the same intensity of exertion. In other words, a high VO_2_max enhances the dominance of aerobic metabolism and raises the anaerobic threshold. This physiological adaptation results in lower blood lactate levels during submaximal exercise in athletes. This suggests that blood lactate levels may serve as an indirect indicator of VO_2_max. Furthermore, data on blood glucose concentration may support the ability to predict VO_2_max. Solomon et al. reported that low aerobic capacity is significantly associated with higher fasting blood glucose concentrations [33]. This physiological relationship means that low VO_2_max performance leads to impaired glycemic control and reduced insulin sensitivity. Based on this mechanism, it is considered that the integration of both pre- and post-exercise glucose concentrations into the VO_2_max prediction model is another important factor that enhances the predictive power of the SVR model. In summary, the fact that the SVR model in the present study predicted VO_2_max performance with a high degree of accuracy may reflect the mathematical relationship between blood lactate and blood glucose concentrations and VO_2_max performance. Moreover, another interesting result of the present research that fills an important void in the available scientific literature is a significant performance gap in the predictive accuracy of the SVR algorithm among genders, presenting a substantially higher degree of predictability for female (R^2^ = 0.890) vs. male participants (R^2^ = 0.702). The cause of such predictive accuracy difference can be attributed to several biological and mathematical reasons. Biologically speaking, as seen in our population’s baseline characteristics, female athletes showed a highly distinctive and uniform, sharp hyperglycemic profile post-test (Δ glucose: 34.15 mg/dL) as opposed to male athletes who had a heterogeneous and stable/hypoglycemic pattern (Δ glucose: −5.43 mg/dL). Generally speaking, women have different substrate kinetics of metabolism, which includes a predominant use of lipids during submaximal exertion, but an acute glucose increase in response to the acute stress of maximal effort associated with catecholamine-driven gluconeogenesis. In terms of machine learning, such sharp and acute metabolic responses coupled with smaller anthropometric variability (e.g., weight) of female athletes made the input feature space tighter and more homogeneous. As a result, the SVR algorithm managed to map the particular metabolic profile into VO_2_max with greater mathematical accuracy than in the case of male participants.

Another original methodological contribution of this study is the presentation of two different models, developed using the same dataset, tailored to different scenario conditions. Whilst the SVR model stands out as a solution for digital platforms requiring high accuracy, taking into account the difficulties arising from the SVR model’s application under field conditions, a highly practical formula known as β-LGE (Beta-Weighted Lacto-Glycemic Equation), based on the beta coefficients of the MLR model, has been developed; VO_2_max = 20.95 + (15.26 × Gender) − (0.10 × Weight) − (0.07 × Pre-Glucose) − (0.06 × Δ Glucose) + (3.43 × Pre-Lactate) + (0.86 × Δ Lactate). Derived from MLR coefficients, this formula has a sensitivity close to that of the SVR model (R^2^ = 0.876) and enables the calculation of athletes’ VO_2_max values with an accuracy rate of 87%.

Many formulas have been developed that prioritize practical application in field trials [34,35,36]. Daros et al. developed two equations to predict VO_2_max performance in young footballers using the maximal field test. The coefficients of determination for these two distance- and speed-based formulas were reported as R^2^ = 0.590 and R^2^ = 0.544, respectively [34]. Although both tests offer advantages in terms of application and cost, they fail to account for approximately 41–46% of the variance. Furthermore, this test requires exercise to be performed to the point of maximum exhaustion. In contrast, whilst the SVR model fails to account for approximately 6% of the variance, the β-LGE formula possesses explanatory power. In another study, Silva et al. developed MLR and artificial neural network (ANN) models to estimate VO_2_max levels in individuals under 18 years of age using a 20-m sports endurance test. The R-value for the MLR method was reported as 0.84, with the standard prediction error reported as 4.90 mL/kg/min; they reported that the VO_2_max prediction formula based on MLR (VO_2_max = 43.313 + 4.567 × sex − 0.560 × BMI + 2.785 × stage) yields better results than both previously published formulas and the ANN [35]. When compared with the findings of this study, it can be said that several key differences and relative advantages have emerged. Although the MLR model has acceptable predictive power (R = 0.84, SEE = 4.9 mL/kg/min), it is seen to be lower than the SVR model obtained from this study (R^2^ = 0.93, RMSE = 1.442 mL/kg/min). Furthermore, the β-LGE formula derived from this study (R^2^ = 0.876) exhibits higher predictive power. The fundamental difference between these findings stems primarily from the differing test requirements and underlying physiological mechanisms of the two approaches. The model based on the 20-m shuttle run requires maximal exertion. This situation carries a risk of injury and may also require a high level of motivation. Furthermore, it may not be suitable for individuals with a low fitness level. In contrast, it can be said that this study offers a more practical model by eliminating the need for exercise habits that require maximal effort.

**Limitations of Study**

This study is limited to individuals aged between 18 and 25. Therefore, this model should be tested on athletes at different stages of development. Furthermore, this model has been developed exclusively for players of protective football. Consequently, the model’s validity should be tested in individual and team sports with different metabolic demands. Metabolic markers such as blood glucose and blood lactate levels may be influenced by nutrition, sleep, circadian rhythm, hydration, and the phases of the menstrual cycle in women. The failure to control these variables may have affected the model’s predictive power. Moreover, even though data augmentation methods proved their statistical efficiency and were used successfully to solve the problem of the limited number of samples, the use of artificial data can be considered a significant limitation of the methodology. The artificial data production technique, which copies physiological limits of the small group of people, might lack the unpredictable elements of physiological noise inherent in diverse real-world populations. Thus, it might contribute to the overstatement of the results of the model and its inability to generalize to more cases. Hence, it is crucial for future research to validate the models by means of large-scale independent empirical data. Additionally, the predictive models developed in this study were not externally validated using an independent sample, which constitutes an important limitation that must be addressed in future research to confirm their widespread reliability. Although the β-LGE formula accurately predicts VO_2_max performance to a high degree, it was found that approximately 12% of the variation is not explained by the model.

## 8. Conclusions

In this study, a dual-purpose and practical machine learning model has been developed that enables the prediction of VO_2_max using only anthropometric measurements and minimally invasive blood glucose and blood lactate tests, without the need for athletes to exert maximum effort. Of the three different models, the SVR model achieved the highest predictive power, whilst the β-LGE formula was developed to facilitate application under field conditions.

Looking ahead, our hypothesis is that including transient biomarkers, like circadian rhythm, nutrition level, sleep quality, and menstrual phase, into this machine learning model would further optimize VO_2_max prediction and allow us to gain insights into how day-to-day metabolic changes influence aerobic metabolism. Moreover, our assumption is that this non-exhaustive biomarker method may also be safely used in a context other than athlete evaluation. In particular, there is a possibility of using the β-LGE model as a user-friendly, stress-free additional way of assessing the initial cardiorespiratory fitness state of regular people. Such usage should not be seen as a diagnostic method but rather as a tool for assessing fitness levels.

**Future research directions**

Future research can compare the values of the calculated VO_2_max with those measured in the laboratory and in field tests, including participants representing a variety of sport disciplines and of different genders and age groups.

**Practical implications**

Although these models demonstrate a high degree of accuracy, due care must be exercised when using them in practice, at least until they have undergone further validation processes on other samples. On the positive side, this approach offers a useful screening tool for practical purposes. Coaches who are unable to measure their players’ VO_2_max values due to a lack of suitable equipment and financial resources should regard this approach as an effective auxiliary tool rather than a definitive clinical tool. Researchers investigating how specific types of training affect athletes’ endurance can also use this new approach to calculate their players’ VO_2_max values. Comparative studies that contrast this digital estimate with actual values obtained in the laboratory will be extremely useful in validating this model.

## Figures and Tables

**Table 1:** Baseline characteristics of participants.

Variable	Overall Median (IQR)	Female Median (IQR)	Male Median (IQR)	*p*-Value
**Age (years)**	21.00 (3.00)	20.00 (2.00)	21.00 (2.00)	<0.001
**Height (cm)**	171.37 (23.15)	159.85 (6.49)	183.09 (7.47)	<0.001
**Weight (kg)**	64.11 (33.25)	55.68 (5.64)	89.11 (13.48)	<0.001
**Pre-Glucose (mg/dL)**	95.91 (22.41)	89.42 (11.57)	109.09 (19.21)	<0.001
**Post-Glucose (mg/dL)**	107.70 (28.05)	122.86 (32.35)	100.44 (16.74)	<0.001
**Delta Glucose (mg/dL)**	6.36 (41.59)	34.15 (36.36)	−5.43 (18.72)	<0.001
**Pre-Lactate (mmol/L)**	1.03 (0.66)	1.07 (0.79)	1.00 (0.54)	0.118
**Post-Lactate (mmol/L)**	11.86 (3.01)	12.11 (2.05)	11.08 (4.00)	0.200
**Delta Lactate (mmol/L)**	10.60 (3.18)	11.12 (2.35)	10.13 (4.02)	0.007
**Max VO2 (mL/kg/min)**	41.02 (11.85)	35.14 (3.73)	47.01 (3.42)	<0.001
**VIFT (km/h)**	15.50 (5.00)	12.50 (1.62)	17.50 (1.00)	<0.001
**HRmax (bpm)**	187.50 (11.00)	187.50 (7.00)	187.50 (16.00)	0.305

**Table 2:** Performance comparison of machine learning algorithms for combined cohort VO_2_max prediction.

Model	R-Squared	RMSE	MAE	MSE	MAPE (%)
**MLR**	0.876	2.103	1.676	4.421	4.17%
**RF**	0.859	2.226	1.659	4.955	4.14%
**SVR**	0.939	1.442	1.097	2.080	2.78%

MLR: Multiple Linear Regression, RF: Random Forest, SVR: Support Vector Regression.

**Table 3:** Comparative performance analysis of ML algorithms for VO_2_max prediction stratified by gender.

Algorithm	Gender	R_Squared	RMSE	MAPE %
**SVR**	Female	0.89	1.065	1.67
**MLR**	Female	0.759	1.541	2.7
**RF**	Female	0.723	1.817	2.86
**SVR**	Male	0.702	1.823	4.25
**MLR**	Male	0.254	2.667	6.1
**RF**	Male	0.214	2.67	5.82

MLR: Multiple Linear Regression, RF: Random Forest, SVR: Support Vector Regression.

## Data Availability

The data supporting the findings of this study are available from the corresponding author upon reasonable request. The data are not publicly available due to privacy and ethical restrictions.

## Abbreviations

The following abbreviations are used in this manuscript:

30-15 IFT	30-15 Intermittent Fitness Test
CPET	Cardiopulmonary Exercise Test
HR	Heart Rate
HRmax	Maximal Heart Rate
QCST	Queens College Step Test
VIFT	Maximum Speed Reached
VO_2_max	Maximal Oxygen Uptake
β-LGE	Beta-Weighted Lacto-Glycemic Equation
